# Supplementary material for: Caloric restriction leads to druggable LSD1-dependent cancer stem cells expansion
Source: Nat Commun. 2024 Jan 27;15:828. doi: 10.1038/s41467-023-44348-y (PMC10821871; doi:10.1038/s41467-023-44348-y)
Supplement: Supplementary file 1 — Supplementary Information [file 41467_2023_44348_MOESM1_ESM.pdf]

Supplementary Information for

Caloric restriction leads to druggable LSD1-dependent cancer stem cells expansion

Pallavi R et al.,

Correspondence to:

Luca Mazzarella, MD, PhD; email: luca.mazzarella@ieo.it

and

Pier Giuseppe Pelicci, MD, PhD; email: piergiuseppe.pelicci@ieo.it

This PDF file includes:
Supplementary Figures 1-10

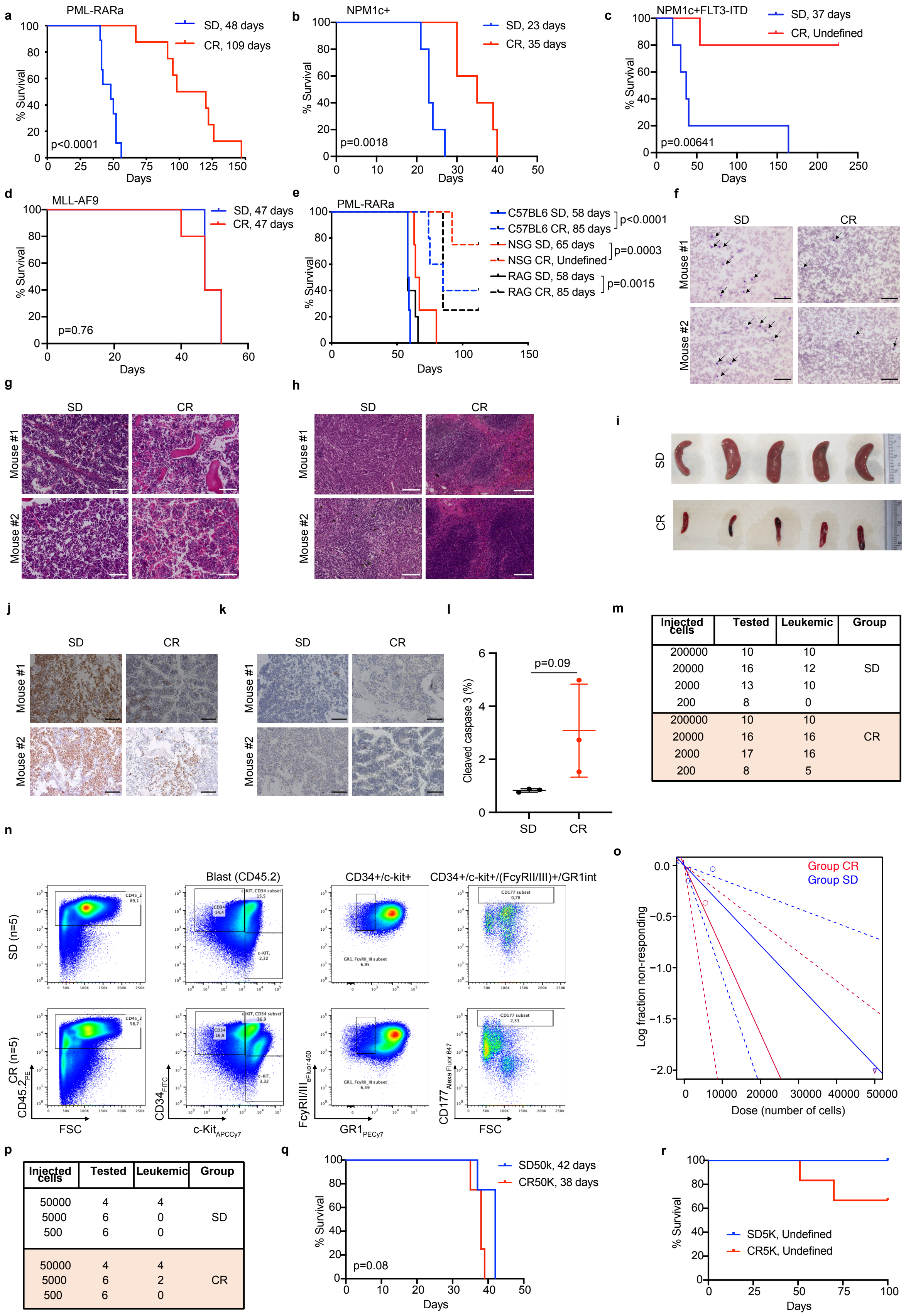

Injected cells	Tested	Leukemic	Group
200000	10	10	SD
20000	16	12	
2000	13	10	
200	8	0	
200000	10	10	CR
20000	16	16	
2000	17	16	
200	8	5	

Injected cells	Tested	Leukemic	Group
50000	4	4	SD
5000	6	0	
500	6	0	
50000	4	4	CR
5000	6	2	
500	6	0	

Supplementary Fig. 1: Anti-leukemic effects of CR on AMLs.

a-d, Kaplan-Meier survival curves of mice injected with: **a,** PML-RARa blasts (2 x 10^6, n=9 (SD) and =8 (CR); The survival curve of PML-RARa is the part of Fig.1a showing the SD and CR_3 group for better comparison here; **b,** NPM1c+ (0.2 x $10^{6;}$ n=5/group); **c,** NPM1c+FLT3-ITD (1 x $10^{6;}$ n=5/group); **d,** MLL-AF9 (1 x 10^6; n=5/group). The average survival in days are indicated. Statistical analysis was performed using log-rank (Mantel-Cox) test; results are indicated at the bottom of the graph. **e,** Kaplan-Meier survival curve of RAG, NSG, and C57BL6 mice injected with APL blasts and subjected to CR. The average survival in days are indicated. *P* value (by Mantel-Cox log-rank test) indicate statistical significance of p=0.0015 in RAG (SD, n=5; CR, n=4), p=0.0003 in NSG (SD, n=4; CR, n=4) and p<0.0001 in C57BL6 mice (SD, n=4; CR, n=5)) for survival under CR. **f,** Representative Giemsa stain of peripheral blood smear for SD (n=2) and CR (n=2) at 4 weeks. **g-h**, Hematoxylin and Eosin staining of bone (**g)** spleen (**h**). **i**, photograph of spleen under SD and CR treatment at 4 weeks. **j**-**k**, IHC staining of ki67 (**j**) or cleaved caspase 3 (**k**) in the BM of APL recipients subjected to SD or CR. **l**, percentage of cleaved caspase 3 in the BM of APL SD or CR recipient by flow-cytometry (n=3 per group; showed in Fig. 3d as a control for better comparison). Data are expressed as mean ± SD. Statistical analysis was performed using two-tailed t-test **m,** Table showing numbers of injected APL blasts from SD and CR mice in the limiting dilution assay, numbers of transplanted mice and numbers of recipient mice that developed leukemia. Data corresponding to number of injected APL blast from 20000 to 200 has been plotted in Fig. 1f, for the better representation **n**, Representative flow cytometry plots showing the gating scheme for Fig. 1g, h and Suppl Fig. 6d for the analyses of the $CD34^+$, c-Kit^+, FcγRIII/II^+, $Gr1^{int}$, $CD177^{high}$ subpopulation. APL blasts were first selected for the CD45.2+ population and then for $CD34^+$/c-Kit^+ population. $CD34^+$/c-Kit^+ sub-population was then selected for expression of FcyRIII/II and intermediate expression of GR1, followed by high expression of CD177, as indicated. **o**, A log fraction plot of limiting dilution model fitted to the data (shown in p) for NPM1c+ using ELDA tool. **p**, Table showing doses of NPM1c+ blasts from SD and CR mice used for limiting dilution assay and the leukemia outcome in the recipient mice. **q-r,** Kaplan-Meier survival curve of C57BL6 mice injected with 50K (**q**) and 5K **(r)** of NPM1c+ blasts sorted from the bone marrow of leukemic SD and CR mice. "n" represents the number of mice/groups. Scale bars: **f-h, j, k** 100 μm. Source data are provided as a Source Data file.

a

SAMPLE	CR 1	CR 2	CR3	SD 1	SD 2	SD 3
Number of total missense/ nonsense mutation	6	4	2	1	4	4
Mean number of missense/ nonsense mutation		4			3	
Mutation frequency per Mbp (number of missense)/ (bases sequenced>=10x)*1Mb]	0.122	0.080	0.040	0.020	0.080	0.080
Number of cancer genes mutated*	0	0	0	0	0	0

b

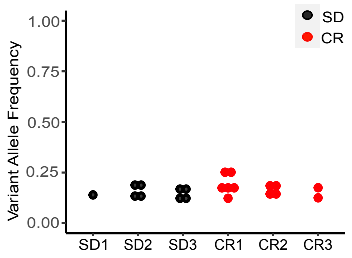

c

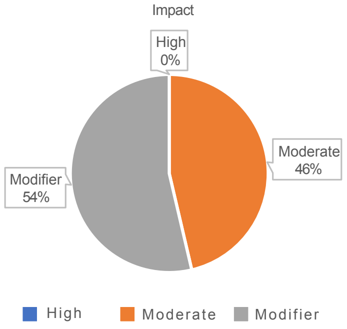

Supplementary Fig. 2: Genomic landscapes of SD and CR APLs.

a, Summary table of SNVs identified by WES in each biological replica for CR and SD conditions (n=3 per group), using the MuTect bioinformatics pipeline. The intersection with the list of Cancer Genes has been done only considering missense and stopgain variants. **b**, Variant allele frequency of DNA mutations identified by WES in each biological replica for CR and SD conditions (n=3 per group). **c,** Pie chart summarizing the variant effect predictor (VEP) result showing the proportion of impact across all the variants. The color scheme of the pie chart is indicated at the bottom. High refers to disruptive impact on the protein, probably causing protein truncation, loss of function or triggering nonsense mediated decay; moderate refers to non-disruptive variant that might change protein effectiveness; modifier refers to non-coding variants or variants affecting non-coding genes. Source data are provided as a Source Data file.

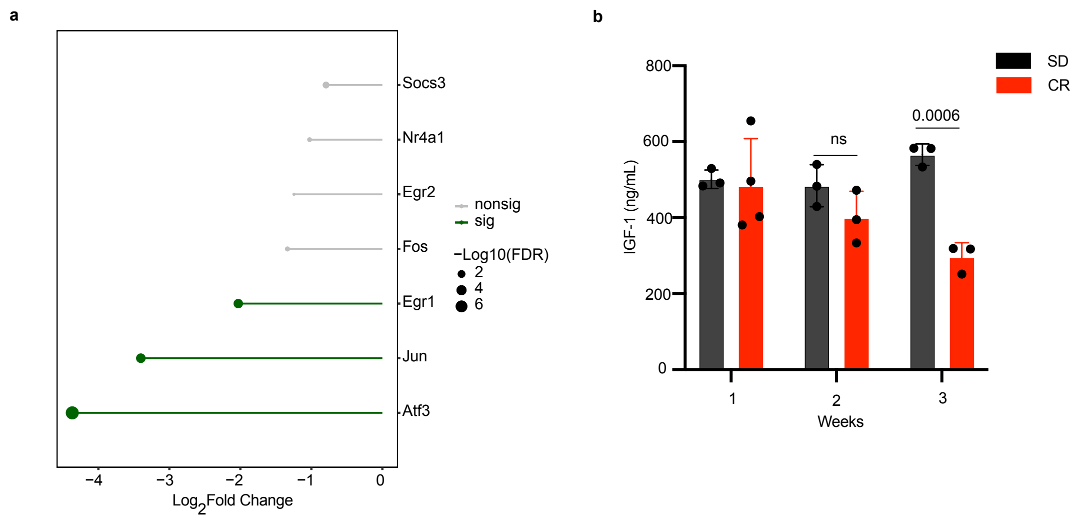

Supplementary Fig. 3: Effect of CR on insulin/IGF1 signaling.

a, Differential expression of selected insulin/IGF1 target genes in CR vs. SD. **b,** Levels over time of circulating IGF1 in the PB of APL recipients in CR and SD (n=3 mouse per condition per time point). "n" represents the number of mice/groups. Data are expressed as mean ± SD. Statistical analysis was performed using two-tailed t-test. Source data are provided as a Source Data file.

a

b

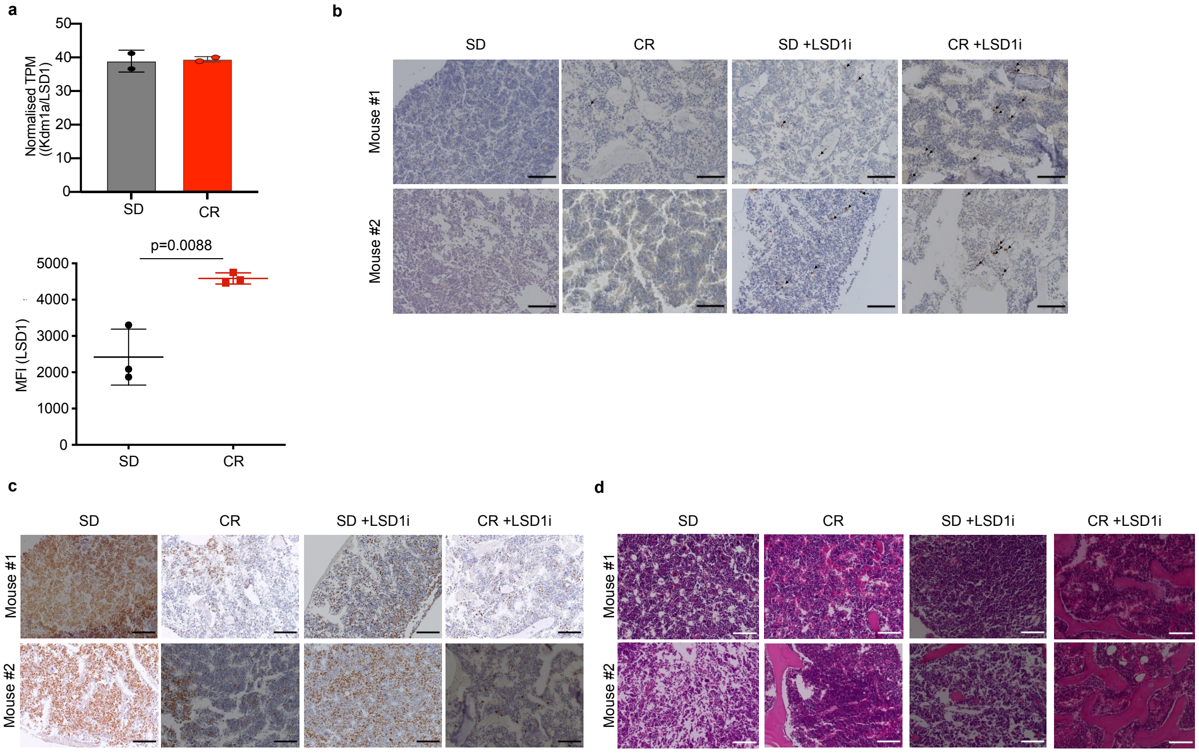

e

Conditions	No. of dead C57BL6, Primary Recipient	No. of Primary Recipient Preserving leukemia Initiation ability in Secondary Transplant (RAG-/-)	Total Leukemic mice	Complete Remission (%)	p- val (2-tailed Fisher test, SD as a reference)
SD	6/6	-	6/6	0	
CR	8/8	-	8/8	0	ns
SD+LSD1i	3/5	1/2	4/5	20	0.4545
CR+LSD1i	1/9	1/5	2/9	77	0.007

f

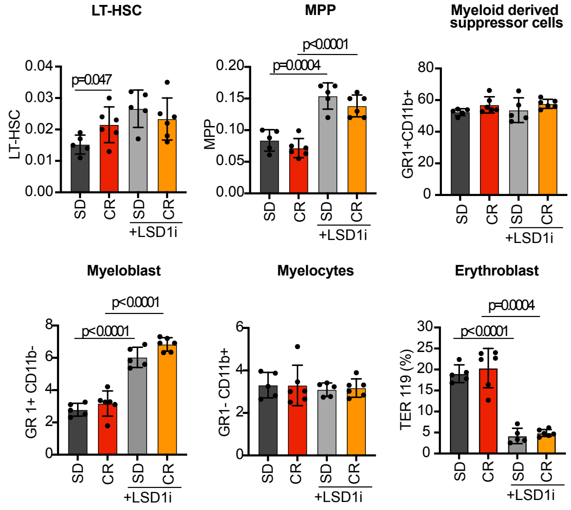

g

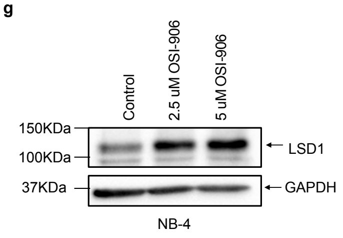

Supplementary Fig. 4: Effect of LSD1 inhibition under normal and CR condition.

a, Normalized LSD1 TPMs (Transcripts Per Kilobase Million) in RNAseq datasets from SD and CR APL sample-replicates (upper); Immunofluorescence FACS-analyses of LSD1 protein levels using anti-LSD1 antibody on BM APL blasts from CR (n=3) or SD (n=3) recipients (lower), sacrificed at 4 weeks after APL injection. Data are expressed as mean ± SD and statistical analysis was performed using two-tailed t-test **b-d,** IHC staining using anti-cleaved caspase 3 (**b**) or anti-Ki67 (**c**) antibodies, or Hematoxylin and Eosin staining (**d**) in the BM of APL recipient mice subjected to SD or CR ± LSD1 inhibitor, sacrificed at 25 days after injection and 4 days after treatment start. **e,** Summary of treatment-outcome in SD- and CR-fed mice transplanted with 2 x 10^6 APL blasts and treated or not with LSD1i, as indicated (first column). The second column reports numbers of dead mice over total numbers of injected mice. The third column shows results of re-transplantation experiments aimed at investigating whether mice that survived upon LSD1i-treatment still carried residual leukemia-initiating cells, as measured by the ability of their BM to generate leukemias in secondary recipients (RAG-/- mouse). 8 x10^6 BM cells were transplanted from each of the indicated LSD1i-treated long-surviving mice. The fourth and fifth column shows the corresponding percent of complete hematological remission, as evaluated by survival of primary (C57BL6 mouse) and the ability of long survived mice to initiate leukemia in secondary (RAG-/- mouse) recipient. The statistical significance was determined by two-tailed Fisher test using SD as a reference. **f,** FACS analyses of HSC/progenitors and differentiated populations (LT-HSC, MPP, Myeloid derived suppressor cells, Myeloblasts, Myelocytes, Erythroblast) in the BMNC of C57BL6 WT mice under CR for 4 weeks and sacrificed after 4 days after LSD1i treatment start (SD, n=5; SD+LSD1i, n=5; CR, n=6; CR+LSD1i, n=6). Data are expressed as percentage of BMNC (bone marrow mononuclear cells) and are mean ± SD. Statistical analysis was performed using two-tailed t-test with Welch's correction. "n" represents the number of mice/ groups for **a**, **e** and **f**. **g**, Western blot analysis for LSD1 expression in NB4 cells (using the anti-LSD1 antibody) upon treatment with the insulin/IGFR1 inhibitor OSI-906, at the indicated concentrations for 24 h. The protein level of GAPDH was used as a loading control (representative of 3 independent experiments). Scale bars: **b-d** 100 µm. Source data are provided as a Source Data file.

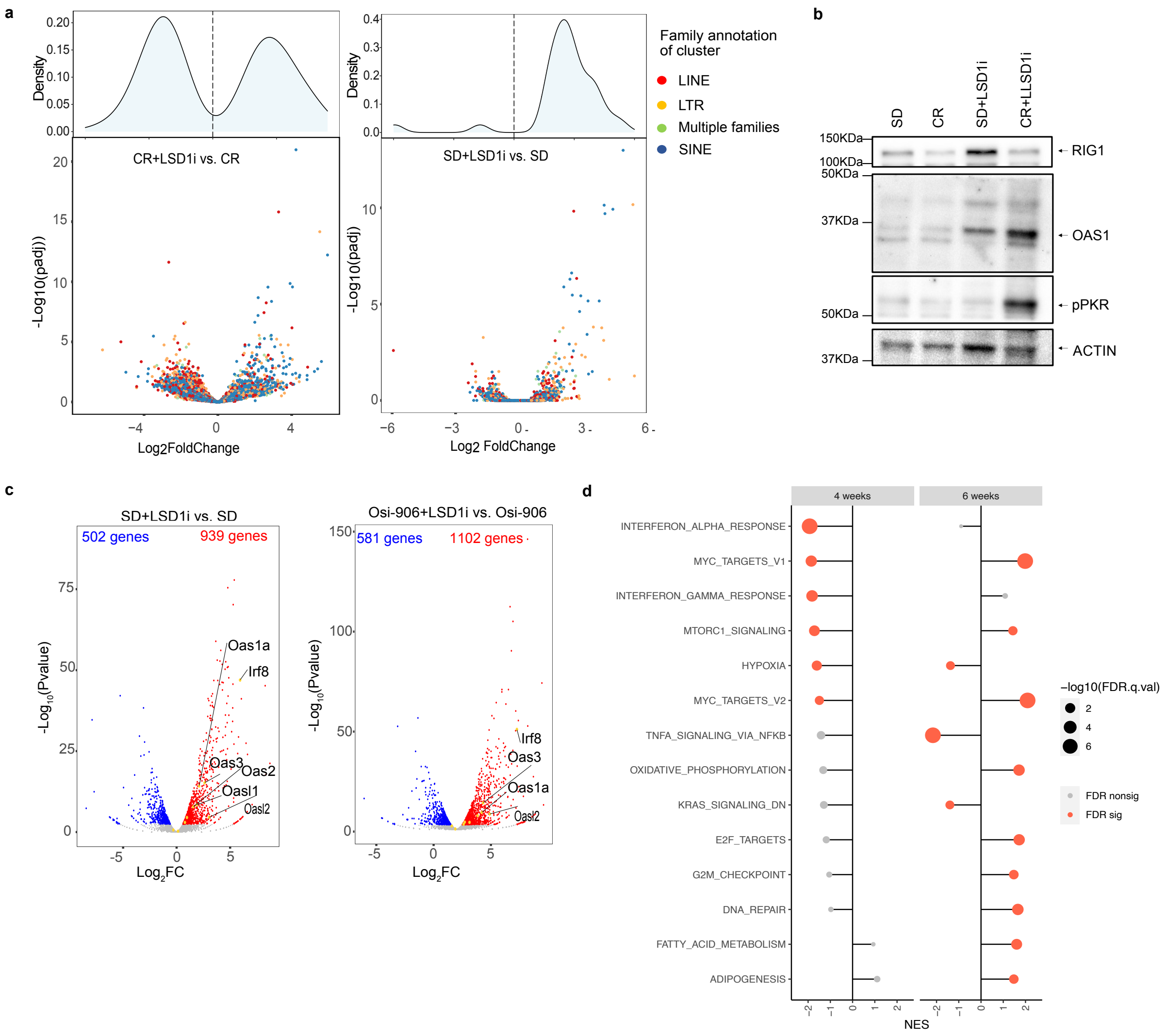

Supplementary Fig.5: Additional analyses of bulk RNAseq datasets.

a, Volcano plots (lower panels) of ERVs differential expression upon LSD1 inhibition in CR (left panel) or SD (right panel). Colored dots have adjusted pVal < 0.05. LINEs, LTRs, SINEs, and multiple families of ERVs are represented by red, orange, blue, and green colors, respectively. Corresponding density plots showing global changes in the expression of ERVs in CR vs. SD are reported on the upper panels. Only ERV families with FDR<0.05 are summarized in the graph **b,** Western blot analyses of key dsRNA-sensing factors (RIG1, OAS1, phosphorylated PKR, as indicated) using corresponding antibodies in APL blasts obtained from mice treated as indicated. Actin was used as an internal control (Representative of two blots). **c,** Volcano plot showing differentially expressed genes in the indicated conditions. Genes in red/blue are significantly up/down regulated (FDR < 0.05). Relevant ISGs are highlighted. Number of upregulated (939 genes in SD+LSD1i vs. SD and 1102 genes in CR+LSD1i vs. CR) and downregulated (502 genes in SD+LSD1i vs. SD and 581 genes in CR+LSD1i vs. CR) genes are reported in the panels for the

comparisons as indicated. **d,** GSEA of bulk RNAseq of samples isolated at 4 or 6 weeks after transplantation, in mice subjected to either SD or CR (n=2 per group; “n” number of mice). Gene sets of the "Hallmark" collection are color-coded based on significance at FDR < 0.05. Only gene sets significant in at least one comparison are shown; sets are ranked based on enrichment at 4 weeks. NES=Normalized Enrichment Score. Source data are provided as a Source Data file.

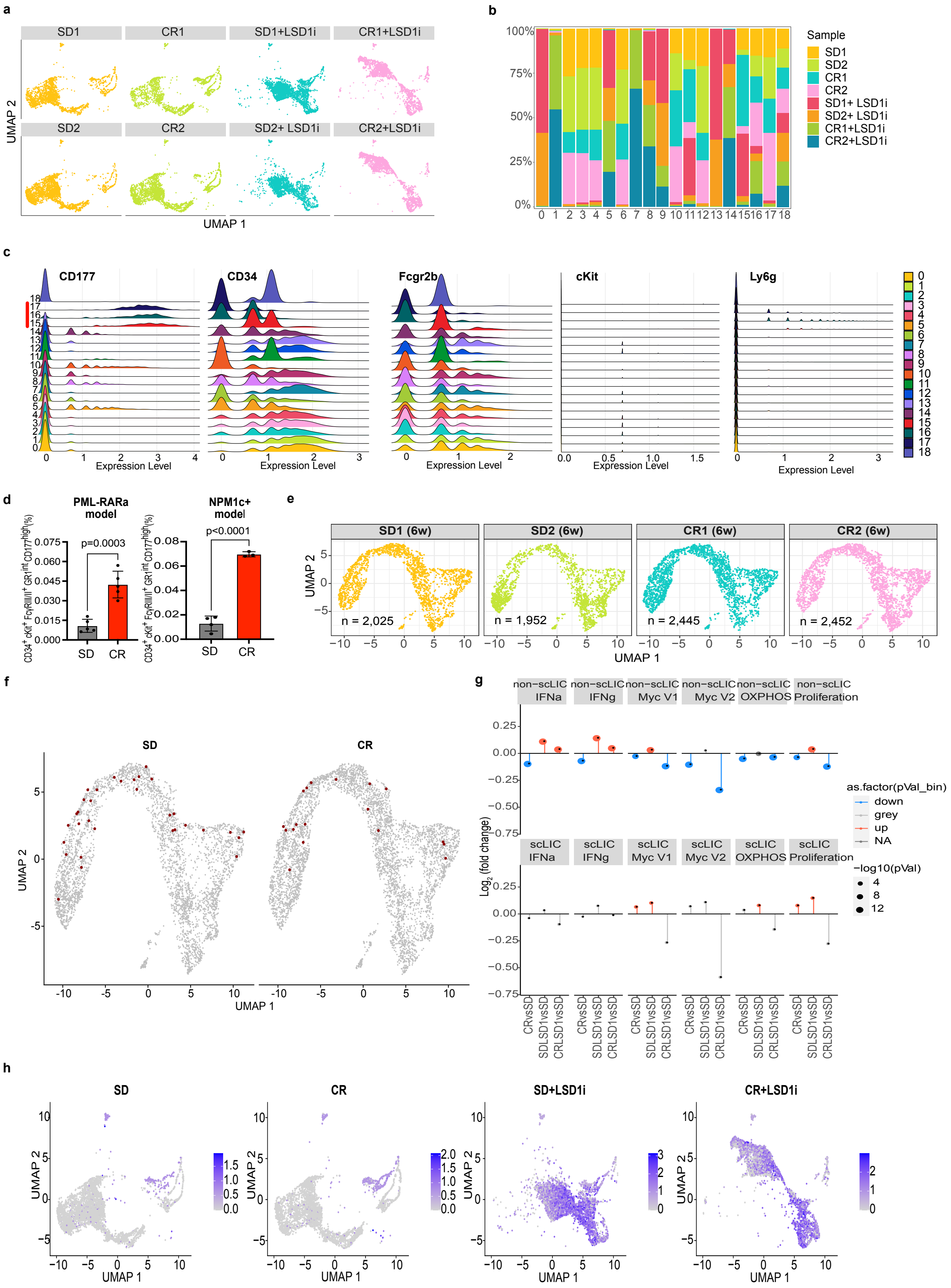

a
SD1
CR1
SD1+LSD1i
CR1+LSD1i
SD2
CR2
SD2+ LSD1i
CR2+LSD1i
UMAP 2
UMAP 1
b
Sample
SD1
SD2
CR1
CR2
SD1+ LSD1i
SD2+ LSD1i
CR1+LSD1i
CR2+LSD1i
c
CD177
CD34
Fcgr2b
cKit
Ly6g
Expression Level
d
PML-RARa model
NPM1c+ model
p=0.0003
p<0.0001
SD
CR
e
SD1 (6w)
SD2 (6w)
CR1 (6w)
CR2 (6w)
n = 2,025
n = 1,952
n = 2,445
n = 2,452
UMAP 2
UMAP 1
f
SD
CR
UMAP 2
UMAP 1
g
non−scLIC IFNa
non−scLIC IFNg
non−scLIC Myc V1
non−scLIC Myc V2
non−scLIC OXPHOS
non−scLIC Proliferation
scLIC IFNa
scLIC IFNg
scLIC Myc V1
scLIC Myc V2
scLIC OXPHOS
scLIC Proliferation
as.factor(pVal_bin)
down
grey
up
NA
−log10(pVal)
CRvsSD
SDLSD1vsSD
CRLSD1vsSD
h
SD
CR
SD+LSD1i
CR+LSD1i
UMAP 2
UMAP 1

Supplementary Fig.6: Additional analyses of scRNAseq datasets.

a, UMAP of cells from two APL biological replicates for each treatment condition (SD, CR, LSD1i and CR+LSD1i), as indicated of scRNAseq data set obtained from the APL blast at 4 weeks post-injection (± LSD1i for 3 days) **b,** Stacked bar charts showing the cluster composition of replicas of each condition (same as in panel a). **c**, Ridge plot reporting expression levels of *Cd177, Cd34, Fcγr2b, c-kit, Ly6g (Gr1)* in each cluster (from 0 to 18). ScRNAseq data set obtained from the APL blast from SD and CR mice at 4 weeks post-injection (± LSD1i for 3 days) **d**, FACS analysis of the $CD34^{+}$, $c\text{-}Kit^{+}$, $Fc\gamma RIII/II^{+}$, $Gr1^{int}$, $CD177^{high}$ sub-population in SD and CR APLs (p=0.0003, SD, n=5; CR, n=5) (left panel)) after 4 weeks post-injection and NPM1c+ AMPLs (p<0.0001, SD, n=4; CR, n=3) (right panel) after 3 weeks post-injection. Statistical analysis was performed using two-tailed t-test. Data are mean ± SD. "n" represents number of mice/ groups. **e-f**, UMAP of scRNAseq datasets from APLs samples at 6 weeks of SD- or CR-treatments (n= 2 mice/ group. "n" number of mice/ groups) (**e**) and distribution of $Cd34^{+}Cd177^{high}$ cells (highlighted in red) in two samples (APL SD; pool of 2 replica and APL CR; pool of two replica) (**f**). **g,** Average fold change (each treatment vs SD as reference) in genes belonging to the indicated Hallmark gene sets in the scRNAseq data, for scLIC and non-scLIC separately**. h**, UMAP plots colored accordingly to *Irf8* expression level among different conditions (SD, CR, SD+LSD1i and CR+LSD1i). ScRNAseq data set of APL blast from SD and CR mice at 4 weeks post-injection (± LSD1i for 3 days). Source data are provided as a Source Data file.

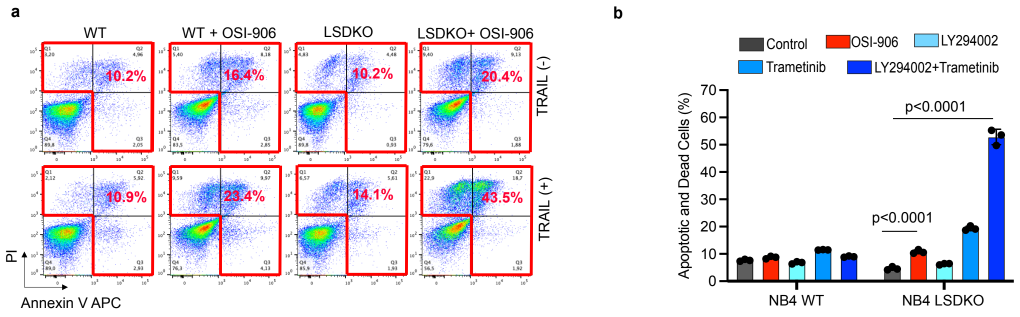

Supplementary Fig. 7: Effects on cell death in cells treated with TRAIL and inhibitors of LSD1, insulin signaling, PI3K or ERK.

a, Gating strategy used for the flow-cytometry analyses for Annexin V and PI stanning for the experiments shown in the Fig 5 a-f, k, Suppl Fig. 7b, Suppl Fig 8b, 8h-i and 8l. FACS plot shown are representative example of gating strategy used, combined with the plot showing the comparison of TRAIL induced cell death in LSD1WT and KO NB4 cells treat with OSI-906 and LSD1 inhibitor as indicated. **b,** Percentage of apoptotic and dead LSD1 WT or KO NB4 cells after treatment with OSI-906 (5 μM), PI3K inhibitor Ly294002 (10 μM), ERK inhibitor Trametinib (4 nM), or the Ly294002/Trametinib combination, in the absence of TRAIL. Data are expressed as mean ± SD (n= 3 biological replicates, representative of 3 independent experiments), two-way ANOVA with post-hoc Tukey's multiple comparison. Source data are provided as a Source Data file.

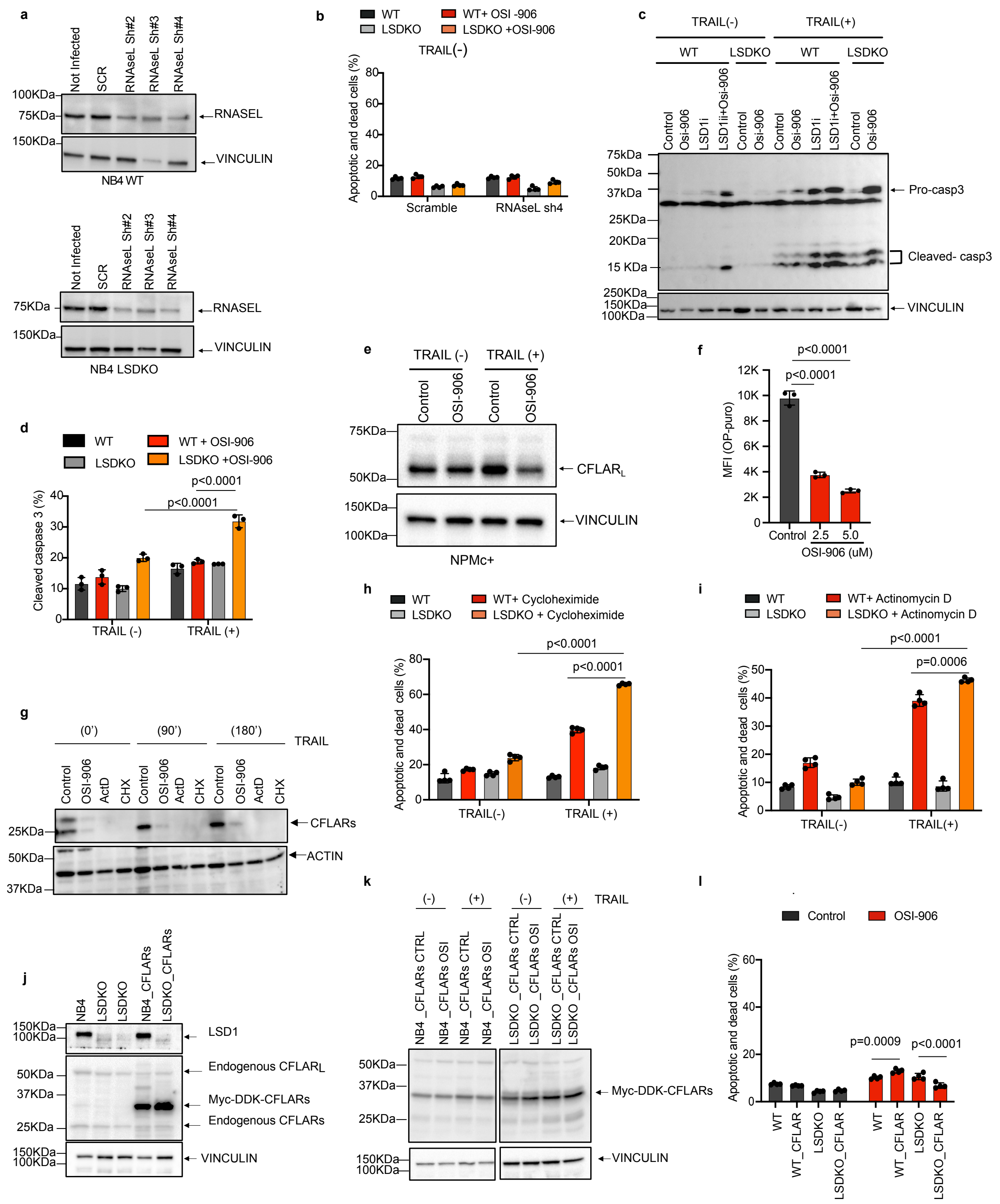

a
Not Infected
SCR
RNAseL Sh#2
RNAseL Sh#3
RNAseL Sh#4
100KDa
75KDa
150KDa
RNASEL
VINCULIN
NB4 WT
NB4 LSDKO
b
WT
WT+ OSI -906
LSDKO
LSDKO +OSI-906
TRAIL(-)
Apoptotic and dead cells (%)
Scramble
RNAseL sh4
c
TRAIL(-)
TRAIL(+)
WT
LSDKO
Control
Osi-906
LSD1i
LSD1ii+Osi-906
Pro-casp3
Cleaved- casp3
VINCULIN
d
WT + OSI-906
LSDKO +OSI-906
p<0.0001
Cleaved caspase 3 (%)
TRAIL (-)
TRAIL (+)
e
TRAIL (-)
TRAIL (+)
OSI-906
CFLAR_L
NPMc+
f
MFI (OP-puro)
OSI-906 (uM)
g
(0')
(90')
(180')
TRAIL
ActD
CHX
CFLARs
ACTIN
h
WT+ Cycloheximide
LSDKO + Cycloheximide
i
WT+ Actinomycin D
LSDKO + Actinomycin D
p=0.0006
j
NB4
NB4_CFLARs
LSDKO_CFLARs
LSD1
Endogenous CFLARL
Myc-DDK-CFLARs
Endogenous CFLARs
k
NB4 _CFLARs CTRL
NB4 _CFLARs OSI
LSDKO _CFLARs CTRL
LSDKO _CFLARs OSI
l
Control
OSI-906
WT_CFLAR
LSDKO_CFLAR
p=0.0009

Supplementary Fig. 8: Effects of RNAseL and CFLAR on TRAIL-induced apoptosis in LSD1 and insulin/IGF1 dual inhibited cells.

a, RNASEL expression upon knockdown by shRNA in LSD1 WT (upper panel) and KO (lower panel) NB4 cells, by WB. **b**, Effect of RNASEL knockdown on cell death (by Annexin V/PI staining) of NB4 LSD1 WT or KO cells ± OSI-906 (5 μM) in the absence of TRAIL (n= 4 biological replicates, representative of 3 independent experiments). **c,** WB of cleaved caspase 3 in LSD1 WT or KO NB4 cells upon TRAIL (100 ng/ml) ± OSI-906 (5 μM) ± LSD1i (2.5 μM). **d,** Apoptosis (by FACS with cleaved caspase 3 staining) of LSD1 WT or KO NB4 cells, ± OSI-906 (5 μM), ± TRAIL (100 ng/ml) (n= 3 biological replicates, representative of 3 independent experiments). **e**, WB of CFLAR expression of NPM1c+ blasts ± OSI-906 (10 μM ± TRAIL (100 ng/ml) for 30 min. Bands corresponding to long ($CFLAR_L$) isoform is shown. **f**, Mean Fluorescence intensity (MFI) of OP-Puro in NB4 cells treated with OSI-906 2.5 μM and 5 μM (n= 3 biological replicates, representative of 2 independent experiments) **g,** WB of CFLAR expression in NB4 cell treated with OSI-906 5 μM, actinomycin D (ActD); 10 ng/ml or cycloheximide (CHX) 1 μg/ml. Bands corresponding to short isoform of CFLAR (CFLARs) is shown. **h-i**, Annexin V/ PI staining on LSD1 WT or KO NB4 cells, treated for 24 h with or without cycloheximide (1 μg/ml) (**h**) or actinomycin D (10 ng/ml) (**i**), ± TRAIL 100 ng/ml (n= 4 biological replicates, representative of 3 independent experiments). **J,** WB for CFLAR expression in LSD1 WT or KO NB4 cells transduced with lentiviruses expressing a Myc-DDK-tagged-$CFLAR_S$, detected using anti-CFLAR antibody. **k**, Effect of OSI-906 and TRAIL on the ectopic expression of Myc-DDK-tagged-$CFLAR_S$ in LSD1 WT and KO cells by WB. **l,** Annexin V/ PI staining on LSD1 WT or KO NB4 cells expressing Myc-DDK-tagged-$CFLAR_S$ without TRAIL (n= 4 biological replicates, representative of 3 independent experiments). Data are shown as mean± SD for **b, d, f, h-i**, and **l**. Two-way ANOVA with post-hoc Tukey's multiple comparison for **b, d, h-i** and **l;** one way ANOVA with post-hoc Tukey's multiple comparison for **f**. WBs are representative of 3 independent experiments; vinculin used as loading control in all blots except in g, where actin was used.

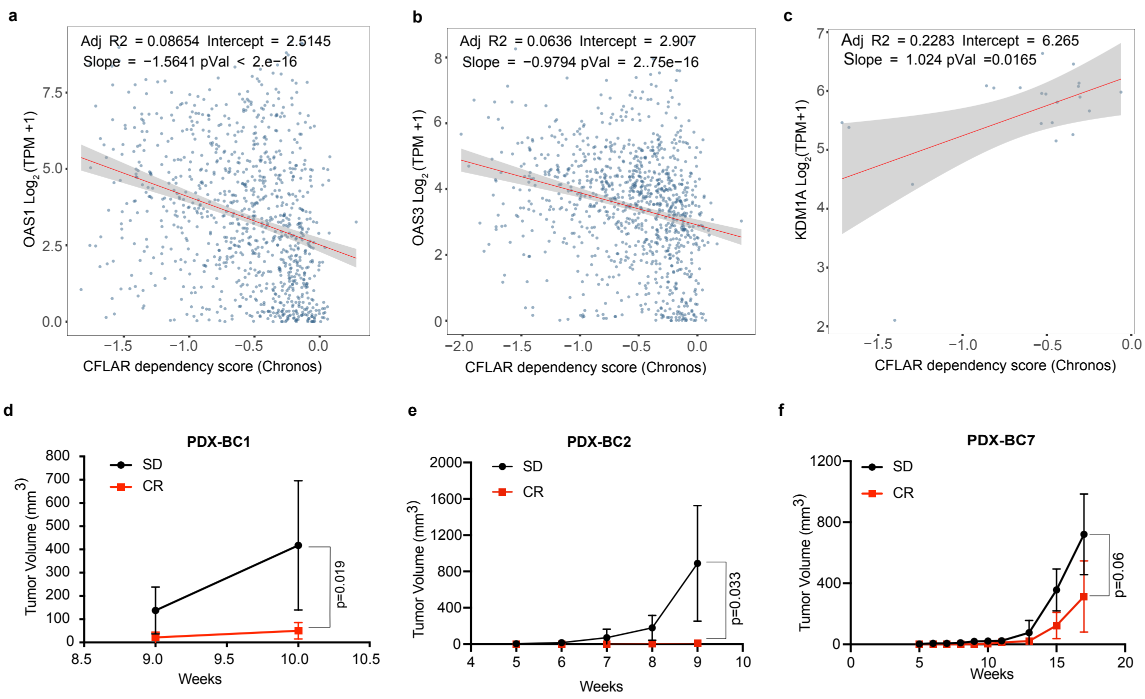

Supplementary Fig. 9: Effect of CR on breast-cancer PDXs.

a-b, DEPMAP analysis of CFLAR dependency vs. OAS1(**a**) and OAS3 (**b**) in all cancer cell lines dataset. **c,** DEPMAP analysis of CFLAR dependency vs LSD1 (KDM1A) in breast cancer cell lines. **d-f,** Effect of CR on growth of three independent TNBC PDX, measured as tumor volume (tumor volume = 1/2 (length X width^2) over time. The tumor sizes were measured with a caliper in two dimensions once every week after the growth of tumors was observed. Data are plotted as mean± SD. n=5 per condition for PDX-BC1. n=4 per condition for PDX-BC2 and PDX-BC7. “n” represents the number of mice/groups. Statistical analysis was performed using two tailed t-test for the last time point. Source data are provided as a Source Data file.

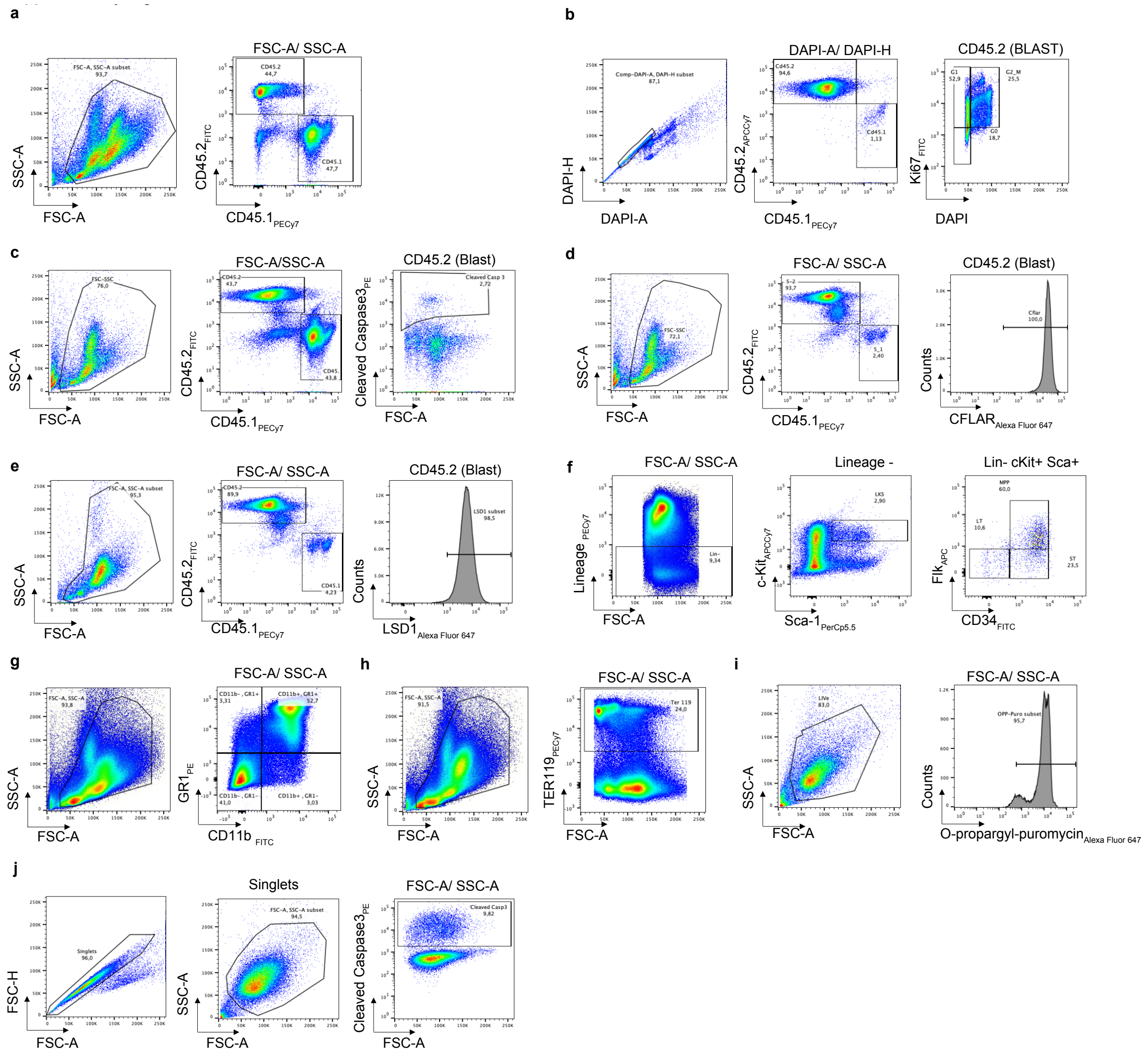

Supplementary Fig 10. Gating strategy used for the flow-cytometry analyses. a, Gating strategy used for the flow-cytometry analyses for the presence of CD45.2 positive APL blast for Fig. 1 b-d and Fig. 3c. **b**, Gating strategy used for the flow-cytometry analyses for cell cycle using Ki67 staining for Fig. 1e. **c**, Gating strategy used for the flow-cytometry analyses for cleaved caspase 3 in the CD45.2 APL blast for the Fig. 3d. **d,** Gating strategy used for the flow-cytometry analyses for level of CFLAR in the CD45.2 positive APL blast for the Fig. 5h. **e**, Gating strategy used for the flow-cytometry analyses for level of LSD1 in the CD45.2 positive APL blast for the Supplementary Fig. 4a. **f,** Gating strategy used for the flow-cytometry analyses in the wildtype bone marrow for the percentage Long Term Haemopoietic Stem Cells (LT-HSC) and multipotent progenitor cells (MPP) for the Supplementary Fig. 4f. **g** Gating strategy used for the flow-cytometry analyses Myeloid derived suppressor cells, Myeloblast, Myelocyte using anti-CD11b and anti-GR1 antibody for the supplementary Fig. 4f. **h**, Gating strategy used for the flow-

cytometry analyses erythroblast using anti-Ter119 antibody for the supplementary Fig 4f. **i,** Gating strategy used for the flow-cytometry analyses O-propargyl-puromycin as a surrogate for the rate of protein synthesis, for the supplementary Fig 8f. **j,** Gating strategy used for the flow-cytometry analyses for cleaved caspase 3 in the NB4 cell lines.
