# Supplementary material for: Caloric restriction leads to druggable LSD1-dependent cancer stem cells expansion
Source: Nat Commun. 2024 Jan 27;15:828. doi: 10.1038/s41467-023-44348-y (PMC10821871; doi:10.1038/s41467-023-44348-y)
Supplement: Supplementary file 3 — Description of Additional Supplementary Files [file 41467_2023_44348_MOESM3_ESM.pdf]

Title: Supplementary Data 1
Description: Variant allele frequency and Variant effect prediction analysis of DNA mutations identified by WES in each biological replica for CR and SD conditions

Title: Supplementary Data 2
Description: Genomic profile and details of breast PDX.
