# Supplementary material for: Caloric restriction leads to druggable LSD1-dependent cancer stem cells expansion
Source: Nat Commun. 2024 Jan 27;15:828. doi: 10.1038/s41467-023-44348-y (PMC10821871; doi:10.1038/s41467-023-44348-y)
Supplement: Supplementary file 6 — Reporting Summary [file 41467_2023_44348_MOESM6_ESM.pdf]

nature portfolio

Corresponding author(s): Luca Mazzarella, Pier Giuseppe Pelicci

Last updated by author(s): Nov 22, 2023

Reporting Summary

Nature Portfolio wishes to improve the reproducibility of the work that we publish. This form provides structure for consistency and transparency in reporting. For further information on Nature Portfolio policies, see our Editorial Policies and the Editorial Policy Checklist.

Statistics

For all statistical analyses, confirm that the following items are present in the figure legend, table legend, main text, or Methods section.

n/a	Confirmed	
☐	☒	The exact sample size (n) for each experimental group/condition, given as a discrete number and unit of measurement
☐	☒	A statement on whether measurements were taken from distinct samples or whether the same sample was measured repeatedly
☐	☒	The statistical test(s) used AND whether they are one- or two-sided<br>*Only common tests should be described solely by name; describe more complex techniques in the Methods section.*
☐	☒	A description of all covariates tested
☐	☒	A description of any assumptions or corrections, such as tests of normality and adjustment for multiple comparisons
☐	☒	A full description of the statistical parameters including central tendency (e.g. means) or other basic estimates (e.g. regression coefficient) AND variation (e.g. standard deviation) or associated estimates of uncertainty (e.g. confidence intervals)
☐	☒	For null hypothesis testing, the test statistic (e.g. F, t, r) with confidence intervals, effect sizes, degrees of freedom and P value noted<br>*Give P values as exact values whenever suitable.*
☒	☐	For Bayesian analysis, information on the choice of priors and Markov chain Monte Carlo settings
☐	☒	For hierarchical and complex designs, identification of the appropriate level for tests and full reporting of outcomes
☒	☐	Estimates of effect sizes (e.g. Cohen's d, Pearson's r), indicating how they were calculated

Our web collection on statistics for biologists contains articles on many of the points above.

Software and code

Policy information about availability of computer code

Data collection

For flow cytometry:
-FACS DIVA v 6.0

Data analysis

FlowJo v10
BWA-MEM (v0.7.8) : http://bio-bwa.sourceforge.net/
GATK (v2.8-1) : https://gatk.broadinstitute.org/hc/en-us
MuTect (v1.1.4) : https://software.broadinstitute.org/cancer/cga/mutect
Annovar (release 2015) : https://annovar.openbioinformatics.org/en/latest/
TopHat (v2.1.0) : https://ccb.jhu.edu/software/tophat/index.shtml
HTSeq (v0.5.3p9) : https://htseq.readthedocs.io/en/master/htseqcount.html
R (v2.15.1, v3.6.2, v4.0.0): https://www.r-project.org/
Gene Set Enrichment Analysis (GSEA) (v3) : https://www.broadinstitute.org/gsea/
ggplot2 (v3.3.5) : https://cran.r-project.org/web/packages/ggplot2/index.html
CellRanger (v4.0.0): https://support.10xgenomics.com/single-cell-gene-expression/software
scDblFinder (v1.1.8) : https://bioconductor.org/packages/release/bioc/html/scDblFinder.html
scran (v1.22.1) : https://bioconductor.org/packages/release/bioc/html/scran.html
ggpubr (v0.4.0) : https://cran.r-project.org/web/packages/ggpubr/index.html
tidyverse (v1.3.1) : https://cran.r-project.org/web/packages/tidyverse/index.html
Seurat (v4.0.6): https://satijalab.org/seurat/
msigdbr (v7.4.1) : https://cran.r-project.org/web/packages/msigdbr/index.html
org.Hs.eg.db (v3.14.0) : https://bioconductor.org/packages/release/data/annotation/html/org.Hs.eg.db.html
Orthology.eg.db (v3.14.0) : http://bioconductor.org/packages/release/data/annotation/html/Orthology.eg.db.html

org.Mm.eg.db (v3.14.0) : https://bioconductor.org/packages/release/data/annotation/html/org.Mm.eg.db.html

For manuscripts utilizing custom algorithms or software that are central to the research but not yet described in published literature, software must be made available to editors and reviewers. We strongly encourage code deposition in a community repository (e.g. GitHub). See the Nature Portfolio guidelines for submitting code & software for further information.

Data

Policy information about availability of data

All manuscripts must include a data availability statement. This statement should provide the following information, where applicable:
- Accession codes, unique identifiers, or web links for publicly available datasets
- A description of any restrictions on data availability
- For clinical datasets or third party data, please ensure that the statement adheres to our policy

The Whole Exome Sequencing, RNA-Seq, ssRNA-Seq and scRNA-Seq generated in this study are deposited at The European Nucleotide Archive (ENA) under the accession number (PRJEB53822) and can be accessed publicly.
Publicly available DEPMAP data (22Q1 Chronos dataset for dependency and the Expression 22Q1 Public dataset for RNAseq) were downloaded from the repository (https://depmap.org/portal/interactive/)
All remaining data is available in the Article, Supplementary and Source Data files
Source data are provided with this paper.

Field-specific reporting

Please select the one below that is the best fit for your research. If you are not sure, read the appropriate sections before making your selection.

[x] Life sciences [ ] Behavioural & social sciences [ ] Ecological, evolutionary & environmental sciences

For a reference copy of the document with all sections, see nature.com/documents/nr-reporting-summary-flat.pdf

Life sciences study design

All studies must disclose on these points even when the disclosure is negative.

Sample size	Sample size was not based on prior specific hypotheses given the exploratory nature of the experiments.<br>A minimum of 4 mice per group was included in order to calculate variance, accounting for possible drop-out due to unrelated causes (e.g. cannibalism)
Data exclusions	No data were excluded
Replication	each in vivo experiment was replicated at least once and each in vitro experiment was conducted in at least triplicate wells. No experiment was excluded from the final analysis
Randomization	mice and cell culture flasks were randomly allocated to the different treatments. No stratification factor was employed
Blinding	The operators were not blind to the allocation because i) mouse experiments could not be blinded since mice allocated to caloric restriction are immediately recognizable; ii) cell culture experiments could not be blinded because flasks need to be labelled in order to be recognized

Reporting for specific materials, systems and methods

We require information from authors about some types of materials, experimental systems and methods used in many studies. Here, indicate whether each material, system or method listed is relevant to your study. If you are not sure if a list item applies to your research, read the appropriate section before selecting a response.

Materials & experimental systems

n/a	Involved in the study	
[ ]	[x]	Antibodies
[ ]	[x]	Eukaryotic cell lines
[x]	[ ]	Palaeontology and archaeology
[ ]	[x]	Animals and other organisms
[x]	[ ]	Human research participants
[x]	[ ]	Clinical data
[x]	[ ]	Dual use research of concern

Methods

n/a	Involved in the study	
[x]	[ ]	ChIP-seq
[ ]	[x]	Flow cytometry
[x]	[ ]	MRI-based neuroimaging

Antibodies

Antibodies used	-CD45.2 monoclonal antibody (104), FITC (eBioscience™; #11-0454-82)<br>-CD45.2 monoclonal Antibody (104), APC (eBioscience™; #17-0454-82)<br>-CD45.2 monoclonal antibody (104), PE (eBioscience™; #12-0454-82)<br>-CD45.2 monoclonal antibody (104), APC-Cy7 (BD Pharmingen™; #560694)<br>-CD45.1 monoclonal antibody (A20), PE-Cyanine7 (eBioscience™; #25-0453-82)<br>-CD45.1 monoclonal antibody (A20), PE (eBioscience; # 12-0453-82)<br>-Alexa fluor® 488 mouse anti-Ki-67 (B56)(BD Pharmingen™; # 558616)<br>-Alexa fluor® 647 Mouse anti-Ki-67(B56) (BD Pharmingen™; #561126)<br>-PE rabbit anti- active caspase-3 (C92-605) (BD Pharmingen™; # 550821)<br>-CFLAR/FLIP (D5J1E) rabbit mAb (Cell signaling; #56343)<br>-CD177 antibody (R&D Systems; #MAB8186)<br>-CD34 monoclonal antibody (RAM34) FITC (eBioscience™ , # 11-0341-82)<br>- FcyRIII/II (CD16/CD32) monoclonal antibody (93), eFluor™ 450 (eBioscience™, # 48-0161-82),<br>-GR1 (Ly-6G/Ly-6C) monoclonal antibody (RB6-8C5), PE, (eBioscience™, # 12-5931-82)<br>- CD117 (c-Kit) monoclonal antibody (ACK2), APC-eFluor™ 780, (eBioscience™, # 47-1172-82)<br>- Sca-1 monoclonal Antibody (D7), PerCP-Cyanine5.5 (eBioscience™, # 45-5981-82)<br>-Flk2 (Flt3/CD135) monoclonal Antibody (A2F10), APC (eBioscience™, # 17-1351-82)<br>-anti-KDM1/LSD1 poly clonal antibody (abcam; # ab17721).<br>-CD11b Monoclonal Antibody (M1/70), PE-Cyanine7 (eBioscience™, M1/70; # 25-0112-82),<br>-CD11b Monoclonal Antibody (M1/70), FITC, (eBioscience™, #11-0112-82)<br>-Ly-6G/Ly-6C Monoclonal Antibody (RB6-8C5), PE-Cyanine7,(eBioscience™, RB6-8C5; # 25-5931-82),<br>-CD3e Monoclonal Antibody (145-2C11), PE-Cyanine7, eBioscience™(eBioscience™, # 25-0031-82),<br>-TER-119 Monoclonal Antibody (TER-119), PE-Cyanine7(eBioscience™, TER119; # 25-5921-82)<br>- CD45R (B220) Monoclonal Antibody (RA3-6B2), PE-Cyanine7 (eBioscience™, RA3-6B2; # 25-0452-82).<br>--BD Pharmingen™ PE Rabbit Anti- Active Caspase-3 (Clone C92-605 (RUO))<br>-anti-Ki67 monoclonal antibody (SP6) (Invitrogen MA5-14520),<br>-anti-cleaved caspase3 monoclonal antibody (Asp175) (Cell Signaling,# 9661)<br>-Caspase-3 polyclonal antibody (Cell Signaling, #9662)<br>-Vinculin (hVin-1), mouse mAb (Sigma-Aldrich; #V9131)<br>-Actin, mouse mAb (AC-40) (Sigma-Aldrich; #A4700)<br>-OAS1 (D1W3A) Rabbit mAb (Cell signaling; #14498)<br>-MDA-5 (D74E4) Rabbit mAb (Cell signaling; # 5321)<br>-RIG-I (D14G6) Rabbit mAb (Cell signaling; #3743)<br>-RNASE L (D4B4J) Rabbit mAb (Cell signaling; #27281)<br>-CFLAR/FLIP (D5J1E) Rabbit mAb (Cell signaling; #56343)<br>-Anti-KDM1/LSD1 poly clonal antibody (abcam; # ab17721)<br>Secondary antibodies used are<br>-alexa fluor 647 affinipure donkey anti-rabbit IgG (polyclonal, Jackson ImmunoResearch Laboratories; #715-605-152)<br>-horseradish peroxidase (HRP)-conjugated anti-mouse IgG (Cell signaling; #7076)<br>- horseradish peroxidase (HRP)-conjugated anti-rabbit IgG (Cell Signaling; # 7074)
Validation	All the antibodies used in this study were previously published and established. They are all commercially available with all the validated by the company. No new antibodies/clones were used in this study.

Eukaryotic cell lines

Policy information about cell lines

Cell line source(s)	NB4 cell lines, derived from a female APL patient, were a kind gift of M Lanotte.<br>Generation of NB4 LSD1 KO cells by CRISPR-Cas9 was previously described (Ravasio et al Science Advances 2020). HEK-293T cell line was acquired from ATCC (RRID:CVCL_0063)and are available in the IEO cell line repository
Authentication	Cell lines are periodically tested for authentication using the ProMega geneprint 10 PCR-based kit (cat n B9510) . Cells are never kept in culture for longer than a month or 10 passages, whichever is shortest
Mycoplasma contamination	Cell lines are periodically tested and for Mycoplasma contamination via the Uphof/Drexler protocol at every new restock, on average every 3 months.
Commonly misidentified lines<br>(See ICLAC register)	no commonly misidentified cell line was used.

Animals and other organisms

Policy information about studies involving animals; ARRIVE guidelines recommended for reporting animal research

Laboratory animals	7–8-week C57BL6J (Ly5.1 and Ly5.2), NOD.Cg-PrkdcscidIl2rgtm1WjI/SzJ (NSG) and RAG-/- mice were purchased from Charles River

	Laboratories Italia and maintained in our animal facility (European Institute of Oncology Cogentech Facility) under specific pathogen-free conditions. Experiments were conducted upon approval of the local Animal Welfare Committee and Ministerial Project no. 1072/15 and 833/18. Specific sexes were used for each experiment, to match the genetic sex of the transplanted tumor. In particular, all mice used as APL recipients were male, to match the sex of the APL transplantable clone. NSG mice used for TNBC PDX were all female.
Wild animals	no wild animals were used in this study.
Field-collected samples	no field collected samples were used in this study.
Ethics oversight	This research work complies with all relevant ethical regulations. All the animal experiments were approved by Italian Ministry of Health (Project no. 1072/15 and 833/18), conducted in accordance with the Italian law and under the control of institutional (European Institute of Oncology) local animal welfare (Cogentech OPBA) and ethical committee.

Note that full information on the approval of the study protocol must also be provided in the manuscript.

Flow Cytometry

Plots

Confirm that:

- [x] The axis labels state the marker and fluorochrome used (e.g. CD4-FITC).
- [x] The axis scales are clearly visible. Include numbers along axes only for bottom left plot of group (a 'group' is an analysis of identical markers).
- [x] All plots are contour plots with outliers or pseudocolor plots.
- [x] A numerical value for number of cells or percentage (with statistics) is provided.

Methodology

Sample preparation	Bone marrow cells, periperal blood, splenocytes obtained after the processing (described in method section) were stained using standard staining protocols. Throughout the processing and staining, cells were always maintained on ice. In brief, cells were washed in staining buffer (2% FBS in PBS), blocked with 5% BSA in PBS for 30 min followed by re-suspension in staining buffer containing fluorochrome-conjugated antibodies. Intracellular staining when required was performed always after cell surface staining. For the intracellular staining cell surface stained BMNC were fixed in Cytofix/Cytoperm buffer, permeabilized in BD cytoperm plus buffer, followed by refixation in Cytofix/Cytoperm buffer. Fixed cells were stained with intracellular antibodies in perm wash buffer 1 hr at room temperature/ overnight at 4 degrees, when required incubated with fluorochrome-conjugated secondary antibody. Following staining, cells were washed and analyzed/ sorted. Similarly, cells from all the in vitro experiments were stained.
Instrument	For flow cytometry: FACS CantoII and FACS Celesta flow cytometer (BD Biosciences, Oxford, UK) were used for multi-parametric and cell-cycle flow cytometry data acquisition. Cell sorting experiments were performed using a FACSAria Fusion cell sorter (BD Biosciences, Oxford, UK).
Software	Analyses were carried out using FlowJo v 9 and 10.
Cell population abundance	Purity of the sorted population was determined by sampling of sorted cells and re-run on the sorter. Purity of the sorter samples was consistently greater than 90%.
Gating strategy	For all the flow cytometry experiment, single stains and FMOs (Full minus one staining) of each fluorophore were used for cytometer setup and compensations. Antibody against CD45.2 was used for selecting a leukemic blast.<br>Specifically: FCS/SSC parameters were set according to mouse BMNC, spleen cells, PBMC and specific cell lines used. Not stained samples were used to set up the negative gate. For in vivo engraftment and leukemic cell (of Ly CD45.2 origin; all the mice leukemia used were originally developed in CD45.2 mice) analysis in the transplanted C57BL6 LyCD45.1 recipient mice, cells (respective BMNC, Spleen or PBMC) from C57BL6 Ly5.2 mice stained with fluorochrome conjugated CD45.2 ab was used to set up the positive gate. In most of the cases differently fluorochrome conjugated CD45.1 was used in addition to fluorochrome conjugated CD45.2 ab to select the leukemic Cd45.2 positive population. For sorting of leukemic population, cells were gated for live cells using the DAPI channel (DAPI-neg) and FSC/SSC, doublets were discriminated by FSC A vs FSC H, CD45.2 and CD45.1 gates were set based on pure spleen cell or BMNC preparations from non-transplanted CD45.2 or CD45.1 mice. In case of viral transduced cell-line, not infected / not transduced cells were used to set the negative gate and GFP positivity was used to select for the infected population.

- [x] Tick this box to confirm that a figure exemplifying the gating strategy is provided in the Supplementary Information.
